# Interdependency of estradiol-mediated ERα activation and subsequent PR and GREB1 induction to control cell cycle progression

**DOI:** 10.1016/j.heliyon.2024.e38406

**Published:** 2024-09-24

**Authors:** M.M. Heldring, B. Duijndam, A. Kyriakidou, O.M. van der Meer, M. Tedeschi, J.W. van der Laan, B. van de Water, J.B. Beltman

**Affiliations:** a Division of Cell Systems and Drug Safety, Leiden Academic Centre for Drug Research, Leiden University, Einsteinweg 55, 2333 CC, Leiden, the Netherlands; b Section on Pharmacology, Toxicology and Kinetics, Medicines Evaluation Board, Graadt van Roggenweg 500, 3531 AH, Utrecht, the Netherlands

## Abstract

Various groups of chemicals that we encounter in every-day life are known to disrupt the endocrine system, such as estrogen mimics that can disturb normal cellular development and homeostasis. To understand the effect of estrogen on intracellular protein dynamics and how this relates to cell proliferation, we aimed to develop a quantitative description of transcription factor complexes and their regulation of cell cycle progression in response to estrogenic stimulation. We designed a mathematical model that describes the dynamics of three proteins, GREB1, PR and TFF1, that are transcriptionally activated upon binding of 17β-estradiol (E2) to estrogen receptor alpha (ERα). Calibration of this model to imaging data monitoring the expression dynamics of these proteins in MCF7 cells suggests that transcriptional activation of GREB1 and PR depends on the association of the E2-ERα complex with both GREB1 and PR. We subsequently combined this ER signaling model with a previously published cell cycle model and compared this to quantification of cell cycle durations in MCF7 cells following nuclei tracking based on images segmented with deep neural networks. The resulting model predicts the effect of GREB1 and PR knockdown on cell cycle progression, thus providing mechanistic insight in the molecular interactions between ERα-regulated proteins and their relation to cell cycle progression. Our findings form a valuable basis to further investigate the pharmacodynamics of endocrine disrupting chemicals and their influence on cellular behavior.

## 1 Introduction

Breast cancer accounts for approximately 24.5 % of all cancer cases and about 15.5 % of cancer deaths worldwide [1]. Breast cancer development might be provoked by long-term exposure to endocrine disrupting chemicals (EDCs) [[2], [3], [4], [5], [6], [7]] that are abundantly present in our every-day life, e.g., in pesticides, plastics, food, personal care products and flame retardants. Because EDCs can mimic or block endogenous hormones, these compounds can disrupt the normal endocrine system [[8], [9], [10]], resulting in adverse outcomes such as unscheduled proliferation, which can culminate in tumor formation [9,11]. Estrogen receptor alpha (ERα) is an important regulator of growth, proliferation and differentiation, and can be activated through binding by several EDCs [[11], [12], [13], [14]]. Therefore, a mechanistic understanding of the regulation of proliferation by ERα is important in the search for a strategy to quantitatively predict the cellular outcome of exposure to EDCs on mammary gland epithelial cells.

ERα, encoded by the *ESR1* gene, is a nuclear transcription factor that can be bound and activated by multiple binding partners. Upon binding of ERα with one of its endogenous ligands, 17β-estradiol (E2), the E2-ERα complex forms homodimers and binds to estrogen response elements (EREs) in the promoters of target genes [[15], [16], [17]]. Activation of target gene transcription requires the binding of coactivators that stabilize the association of ERα with the DNA and stimulate transcription [18,19]. However, the requirement of coactivator binding differs between genes [19]. Among the well-known estrogen-regulated genes are growth-regulating estrogen receptor binding 1 (gene name *GREB1*; protein name GREB1), progesterone receptor (gene name *PGR*; protein name PR) and trefoil factor 1 (gene name *TFF1*; protein name TFF1). Interestingly, the GREB1 protein functions as coactivator for ERα [20], although it is not known whether only specific genes are dependent on GREB1 binding. PR belongs to the same family of nuclear steroid hormone receptors as ERα and is activated upon binding to progesterone. Apart from its transcriptional activity, PR can bind ERα in the presence of progesterone [21,22], which suppresses the transcriptional activity of ERα and has an antiproliferative effect [[23], [24], [25]]. PR transfection also has a ligand-independent antiproliferative effect on E2-stimulated cells [26]. In contrast, unliganded PR-B (i.e., one of the PR isoforms) can form a complex with ERα and other protein kinases, which binds EREs to promote target gene transcription and enhance proliferative responses to estradiol [27]. Thus, the crosstalk between ERα and PR is important in the regulation of target gene expression and proliferation. Much less is known about the biological role of TFF1, but it can serve as diagnostic [28] and prognostic biomarker for breast cancer. TFF1 expression is thought to protect against breast cancer [29] and high expression levels in breast cancer patients are associated with a good prognosis [30,31]. However, conflicting studies showed enhanced migration [32] and tumorigenesis in mammary tissue upon expression of TFF1 [33]. Thus, considerable controversy exists about the role of TFF1 in breast cancer.

In addition to their transcription regulatory function, ERα, GREB1, PR and TFF1 also play a role in regulation of proliferation. Proliferating cells enter the cell cycle in growth phase 1 (G1). After passing the G1 restriction point, cells start DNA synthesis in the S phase and subsequently enter growth phase 2 (G2). In the final cell cycle stage, cells are preparing for mitosis and divide (M). Transitions through these cell cycle phases are regulated by the physical association between cyclins and cyclin-dependent kinases (CDKs). In response to estrogen, ERα, GREB1, PR and TFF1 each contribute to cell cycle progression in their own ways. First, ERα transcriptionally activates cyclin D1 that binds and activates CDK4/6, stimulating G1 progression [[34], [35], [36], [37]]. Specifically, the cyclin D1-CDK4/6 complex binds and hyper-phosphorylates the tumor suppressor protein retinoblastoma (pRB) [38], which promotes the dissociation of pRb from transcription factor E2F [39] and subsequent transcription initiation of target genes involved in cell cycle progression [[40], [41], [42]]. Second, ligand-activated PR initiates transcription of target genes involved in proliferation by direct interaction with DNA, but also induces gene expression through association with other transcription factors [43]. For example, ligand-bound PR can activate cyclin D1 transcription in the presence of ERα [22,43]. Moreover, PR can directly activate cytoplasmic signaling pathways, such as the cell cycle regulatory PI3K/Akt/mTOR [43] or the MAPK pathway [44]. Third, GREB1 influences cell cycle progression in an ERα-dependent and independent manner. Specifically, ERα stimulation by estrogen leads to activation of the PI3K/Akt/mTOR pathway via GREB1 [45]. In addition, GREB1 knockdown and exogenous GREB1 expression cause an ERα-independent decrease in proliferation [46,47]. Finally, TFF1 is negatively associated with cell proliferation [48,49] by increasing the expression of CDK inhibitors that impede E2F functionality [50]. In contrast, we previously demonstrated a significant reduction of proliferation in ERα-positive MCF7 human breast cancer cells after siRNA-mediated knockdown of TFF1, similar to the effect of ERα, GREB1 or PR knockdown [51]. Despite these multiple studies focusing on the roles of ERα, PR, GREB1 and TFF1 in cell cycle progression, the full mechanisms behind ERα-mediated cell cycle regulation, and especially their quantitative impact remains to be elucidated.

To gain quantitative insight in the interdependencies between protein expression in the ERα signaling pathway and their effect on the cell cycle, we adopted a mathematical modeling approach. The E2-dependent induction of ERα downstream targets has not been modeled previously, although mathematical models related to estrogen signaling do exist. One such study focused on the switch between estrogen and growth factor signaling, and E2-independent cancer cell growth [52]. Another modeling study used direct estrogen supply as stimulant for tumor cell growth, which determined the relation between E2 and tumor growth [53]. In addition, mathematical models of the cell cycle are ubiquitous and differ greatly in complexity, with respect to either protein level or cell cycle phase level (e.g. Refs. [[54], [55], [56], [57], [58], [59], [60]]). As one of the many interesting examples, the simplified cell cycle model by Ferrell et al. (2011) [57] was subsequently used for cell phase classification by Bae et al. (2019) [58]. Importantly, these studies do not yet provide quantitative insight into the relation between ERα-regulated protein expression and cell cycle progression. Constructing a model that describes this relation can help to understand the mechanisms underlying adversity and can ultimately be incorporated into quantitative adverse outcome pathways (qAOPs), which establish quantitative relationships between key events and ultimately lead to defined adverse outcomes [61,62].

Using ordinary differential equation (ODE) modeling, we here aimed to find a mechanistic explanation for observations of protein expression and cell cycle progression in MCF7 cells derived from live-cell confocal microscopy data. For this purpose, we exploited MCF7 BAC-GFP reporter cell lines for GREB1, PR and TFF1, in addition to an MCF7 fluorescent ubiquitination-based cell cycle indicator (FUCCI) cell line [51]. Based on time-resolved protein expression and cell cycle progression data at single cell level, we propose a novel E2 signaling model that connects to a simplified cell cycle model by Ferrell et al. (2011) [57]. Our model suggests that transcriptional activation of GREB1 and PR is not only dependent on association of the E2-ERα complex with GREB1, but also with PR. In addition, we show that this model can be combined with an elementary cell cycle model of two oscillating proteins to qualitatively predict changes in cell cycle progression in response to siRNA-mediated molecular manipulation.

## 2 Results

### 2.1 An ODE model for E2 signaling provides insight into protein-protein interactions

**Fig. 1:**
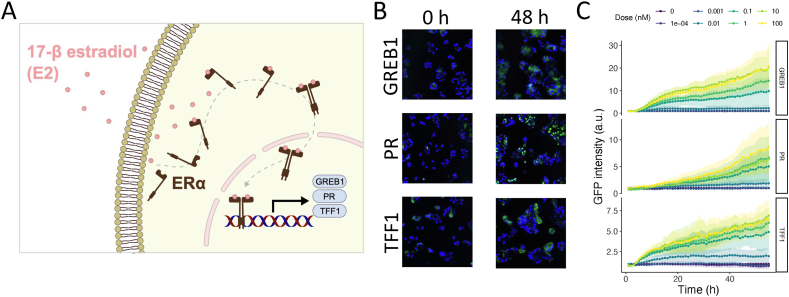
**E2-induced protein expression.** A) Transcriptional activation of GREB1, PR and TFF1 in response to E2 binding to the ERα receptor. B) Example images for the MCF7 cell nuclei (Hoechst staining, blue) and protein expression (GFP, green) at 0 and 48 h after exposure to 100 nM E2. GREB1 and TFF1 are mainly expressed in the cytoplasm, whereas PR is only detectable in the nucleus. C) Dynamics of quantified protein expression at different E2 concentrations. Mean ± SD of three biological replicates.

Binding of E2 to the ERα receptor and subsequent dimerization leads to transcriptional activation of downstream targets GREB1, PR and TFF1 (Fig. 1A). Knockdown experiments in MCF7 cells have highlighted the interdependencies of these proteins and their relevance in cell cycle regulation in a qualitative manner [51]. To obtain the detailed dynamics of ERα target expression at protein level over a period of more than two days, we monitored the induction of GFP-labelled GREB1, PR and TFF1 proteins with live-cell confocal microscopy (Fig. 1B; blue indicates nuclei and green indicates expression of respective protein) at different E2 concentrations using earlier established reporter cell lines [51]. E2 exposure induced protein expression in a concentration- and time-dependent manner (Fig. 1C). GREB1 and TFF1 demonstrated a biphasic expression pattern, with a rapid increase in the first 15 h and a slower but sustained increase in the following hours. PR dynamics were qualitatively different, with a sustained increase over the entire measurement period of 55 h.

**Fig. 2:**
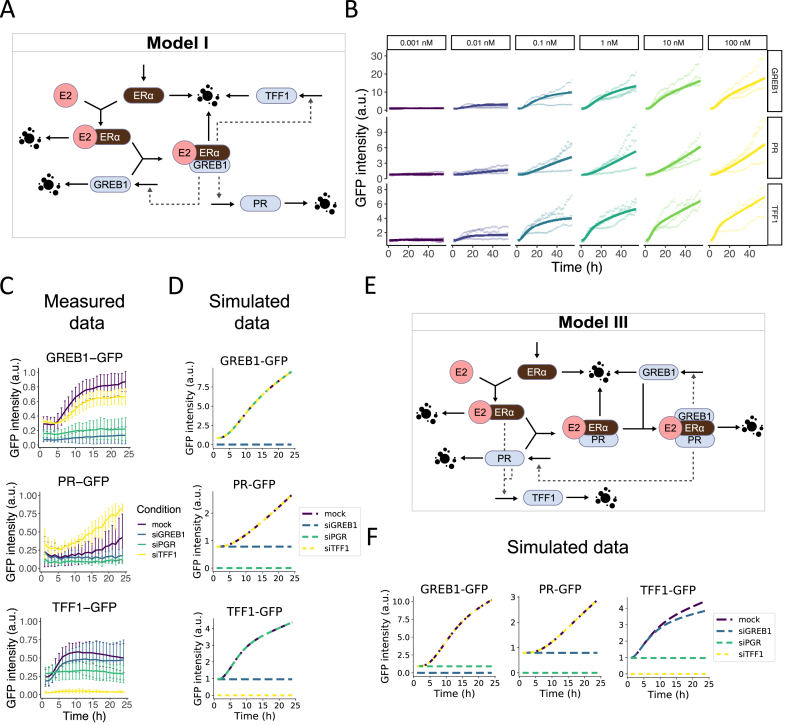
**E2 signaling models and predictions of protein-protein interaction dynamics.** A) Schematic diagram of the E2 signaling model I. E2 and ERα form an E2-ERα complex that can subsequently bind GREB1 to stimulate synthesis of GREB1, PR and TFF1. Solid arrows: synthesis and degradation; dashed arrows: modulation. B) Model simulations for Model I after parameter calibration (solid lines) to the experimental data (points, three independent replicates separately shown). C) Data from protein dynamics in control situation (mock) or after siRNA-mediated knockdown, followed by 24 h exposure to 1 nM E2 (mean ± SD of three biological replicates). D) Predictions of GREB1, PR and TFF1 expression after complete protein knockdown in Model I. E) Schematic diagram of the E2 signaling model III (for model II refer to Supplementary Fig. 1). E2 and ERα form an E2-ERα complex that can also bind PR. E2-ERα and PR stimulate the synthesis of TFF1. E2-ERα/PR can bind GREB1 and the resulting E2-ERα/PR/GREB1 complex stimulates GREB1 and PR synthesis. Solid arrows: synthesis and degradation; dashed arrows: modulation. F) Simulations of GREB1, PR and TFF1 expression after complete protein knockdown in Model III.

To mathematically describe the interactions between ERα and its downstream targets in response to estrogen exposure as reported in literature, we developed an ODE model for the ERα signaling pathway (Model I; Fig. 2A; for details see Methods). In this model, binding of E2 with ERα and subsequent binding to GREB1 [20] leads to transcriptional activation of GREB1, PR and TFF1. We calibrated the model parameters to the experimental data for protein expression dynamics. Importantly, model simulations matched the data very well quantitatively, and they also mirrored their qualitative characteristics, i.e., bi-phasic GREB1 and TFF1 dynamics, and sustained PR activation (Fig. 2B; Supplementary Fig. 1A). In addition, the model generates insight in the mechanism behind the biphasic expression pattern of GREB1 and TFF1. The rapid binding of E2 to ERα leads to fast formation of this complex during the initial phase after E2 exposure, yet also quickly depletes free E2 and ERα (Supplementary Fig. 1A). This depletion slows down the formation of a larger complex of E2-ERα with GREB1 during the second phase. Because this large complex drives protein expression, GREB1 and TFF1 follow the biphasic pattern of this complex. Surprisingly, PR expression increases almost linearly, similar to the measured protein expression pattern, which is due to relatively low value for the maximal E2-ERα/PR/GREB1-dependent stimulation rate of PR.

To assess the predictive capacity of our model for the expected relations between the ERα targets at protein level, we used previously published knockdown data [51] (Fig. 2C). These data demonstrated a dependency of GREB1 on PR availability and vice versa, and a dependency of TFF1 expression on the presence of PR, but not GREB1. As can be expected from its structure, our simple literature-based model is not sufficient to explain the dependencies of GREB1 and TFF1 on PR. Indeed, simulation of complete knockdowns of the proteins did not fully match the experimental data (Fig. 2D). To make GREB1 and TFF1 dependent on PR, we adjusted the model by including PR in the E2-ERα/GREB1-dependent stimulation of GREB1 and TFF1 (Model II; Supplementary Fig. 1B). This model fitted the expression dynamics upon E2 exposure approximately equally well as model I, including the biphasic dynamics of GREB1, TFF1 and E2-ERα/GREB1 complex due to fast depletion of E2 and ERα initially (Supplementary Figs. 1C–D). However, the interdependencies of the proteins revealed after knockdown were still not completely reproduced, because GREB1 knockdown is erroneously predicted to lead to strong TFF1 overexpression (Supplementary Fig. 1E). Therefore, we created a third model, in which we included PR binding to E2-ERα following the experimental evidence for such binding by Daniel et al. (2016) [27], prior to the association with GREB1 (Model III; Fig. 2E). In this model, the E2-ERα complex and PR jointly promote transcriptional activation of TFF1. Moreover, the E2-ERα/PR/GREB1 complex stimulates GREB1 and PR. Calibration of this model gave an equally good fit to the E2 exposure data as models I and II, including the biphasic dynamics of GREB1, TFF1 and intermediate complexes E2-ERα/PR and E2-ERα/PR/GREB1 due to fast E2 and ERα depletion in the initial phase (Supplementary Fig. 1C and Supplementary Fig. 2A). Note that we performed calibration of this model with 75 distinct initial parameter sets, which led to six different optima (top 3 shown in Supplementary Fig. 2B). The second best optimum (61 fits) reproduced the biphasic dynamics slightly better than the optimum with the lowest cost (8 fits), hence we continued with the second best optimum. Because the parameter sets within this optimum were highly similar (Supplementary Fig. 2C), we chose an arbitrary parameter set from these 61 fits to continue our study. Importantly, simulation of complete knockdowns of GREB1, PR and TFF1 in this model qualitatively matched the knockdown data (Fig. 2F). Simulations covering a long time period after E2 exposure showed recovery of protein expression for GREB1 and PR upon E2 disappearance, but not TFF1 (Supplementary Fig. 2D). This suggests that crucial information for correct predictions of long-term TFF1 decay may be lacking. Nevertheless, our model suggests that physical association of PR to the E2-ERα complex is necessary to explain the ERα target expression data at protein level and their interdependencies.

### 2.2 Integration of the E2 signaling model with a cell cycle model predicts cell cycle progression

**Fig. 3:**
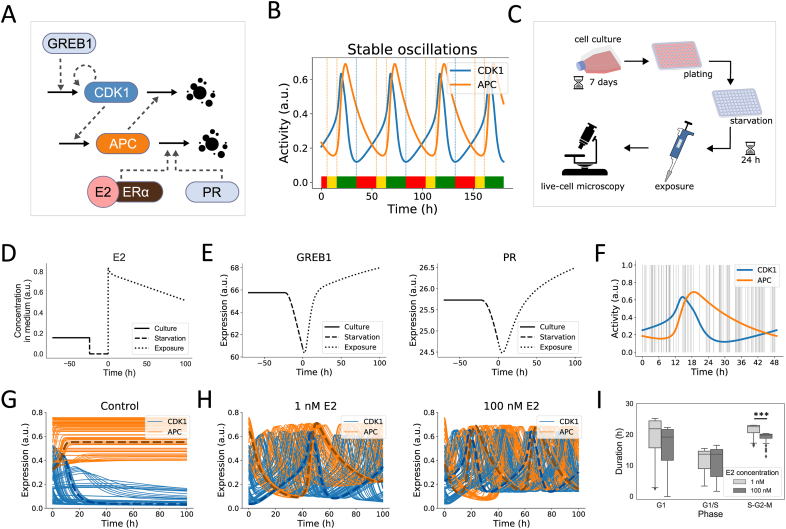
**Model simulations of cell cycle progression with interference of E2-ERα, GREB1 and PR.** A) Schematic representation of the cell cycle model with active forms of CDK1 and APC, and the effect of E2-ERα, GREB1 and PR on these activities. B) Simulations of oscillations of CDK1 and APC activities under constant E2 availability, i.e., when ERα-signaling proteins are in steady state. The colored bar indicates the assigned cell cycle phases (red, G1; yellow, G1/S transition; green, S-G2-M). C) Flow of subsequent experimental phases used to reproduce the experiment *in silico*. D-E) Simulated trajectory of E2 concentration (D) and of GREB1 (E, left) and PR (E, right) expression in the subsequent culture, starvation and 100 nM E2 exposure phases. F) Hundred randomly chosen CDK1 and APC initial activities from one full cell cycle (indicated by vertical lines). G-H) Simulations of CDK1 and APC activities in 100 unsynchronized cells during the first 100 h in control condition, i.e., without E2 stimulation (G), or after 1 or 100 nM E2 exposure (H). Typical time traces have been highlighted for clarity (dashed lines; dark orange, APC; dark blue, CDK1). I) Quantification of *in silico* cell phase durations for the trajectories in H. ∗p < 0.01; ∗∗p < 0.001, ∗∗∗p < 0.0001.

Previous research has highlighted the influence of GREB1, PR and TFF1 on cell cycle progression, i.e., knockdown of these proteins shifts the proportion of cells in G1, G1/S and S-G2-M cell cycle phases [51]. We aimed to study this relationship in more detail by quantifying the influence of E2, GREB1 and PR on cell cycle phase duration. For this purpose, we combined our E2 signaling model with an existing cell cycle model that simulates the oscillations of active forms of CDK1 and APC during the cell cycle [57,58] (Fig. 3A). Although more complex models exist that describe cell cycle dynamics more accurately, we adopted the approach by Bae et al. [58], because of the explicit connection between protein activity and cell cycle phases. Note that we did not include the potential contribution of TFF1 to the cell cycle, due to the absence of TFF1 decay in our simulations at late timepoints. Because CDK1 is indirectly stimulated by GREB1 through GREB1-dependent activation of Akt [45] that in turn inactivates the CDK inhibitor p21 [[63], [64], [65]], we made the activation of CDK1 dependent on GREB1. Moreover, E2 and PR stimulate progression through the G1 phase via production of cyclin D1 [[35], [37], [44], [46]]. To accelerate the transition from G1 to S-G2-M, we made the inactivation of APC dependent on the availability of the E2-ERα complex and PR. Depending on the strength of these interactions, constant availability of E2 resulted in sustained oscillations between active CDK1 and active APC (Supplementary Fig. 3; see Methods for details), from which we determined cell cycle phase durations (Fig. 3B; red indicates G1, yellow G1/S transition, and green S-G2-M phase). In a previous study, the minimum of active CDK1 defined the transition from S-G2-M phase to G1, i.e., green to red, and the minimum of active APC defined the transition point between G1 and S-G2-M [58]. Because we also aimed to determine the duration of the G1/S transition phase, i.e., the yellow phase, we modified the second of the previously used transition rules. Instead of basing the transition on the minimum of active APC, we defined a threshold for active APC: when the fraction of active APC decreased below this threshold, cells went from G1 into G1/S transition phase and when that fraction increased above the threshold, cells progressed from G1/S into S-G2-M phase. For the transition from S-G2-M phase to G1, we followed the previously proposed minimum of active CDK1 as transition point.

To generate predictions for E2-stimulated cell cycle progression, we mimicked the experimental steps (culturing, plating, starvation and exposure) *in silico* (Fig. 3C). Experimentally, cells were cultured in complete medium containing growth stimulatory compounds that have estrogenic properties. We estimated the concentration of E2-equivalent compounds in complete medium based on the degradation rate of GREB1, PR and TFF1 in starvation medium without addition of E2 (Supplementary Fig. 4; see Methods for details), and used this concentration to simulate the steady state protein concentrations during culture conditions. Following steady-state attainment during *in silico* culture (Fig. 3D, solid line), we simulated the 24-h starvation period, during which E2 was removed from the system (Fig. 3D, dashed line). Right after 100 nM E2 exposure, we considered E2 to peak instantly, after which E2 levels biphasically dropped towards baseline levels (Fig. 3D, dotted line). In our simulations, removal of E2 during starvation led to a drop in the expression of GREB1 and PR, but the proteins recovered quickly after exposure (Fig. 3E). In addition, we simulated CDK1 and APC activity under different E2 exposure conditions for 100 unsynchronized virtual cells, by choosing 100 random starting positions during one full cell cycle (Fig. 3F). Without E2, cells rapidly entered a cell cycle arrest in G1, indicated by the sustained low abundance of active CDK1 after a CDK1 activity minimum, i.e., after mitosis (Fig. 3G). After E2 exposure, cell cycle progression increased and the virtual cells had multiple CDK1-APC oscillation cycles (Fig. 3H). Quantification of cell cycle phase durations in our simulated cells exposed to 1 and 100 nM E2 indeed demonstrated a small decrease in G1 (albeit not significant) and a decrease in S-G2-M phase durations, but not for G1/S phase (Fig. 3I). Thus, our combined E2 signaling and cell cycle model can describe faster cell cycle progression upon E2 exposure.

### 2.3 E2 exposure, GREB1 and PR affect cell cycle phase durations

**Fig. 4:**
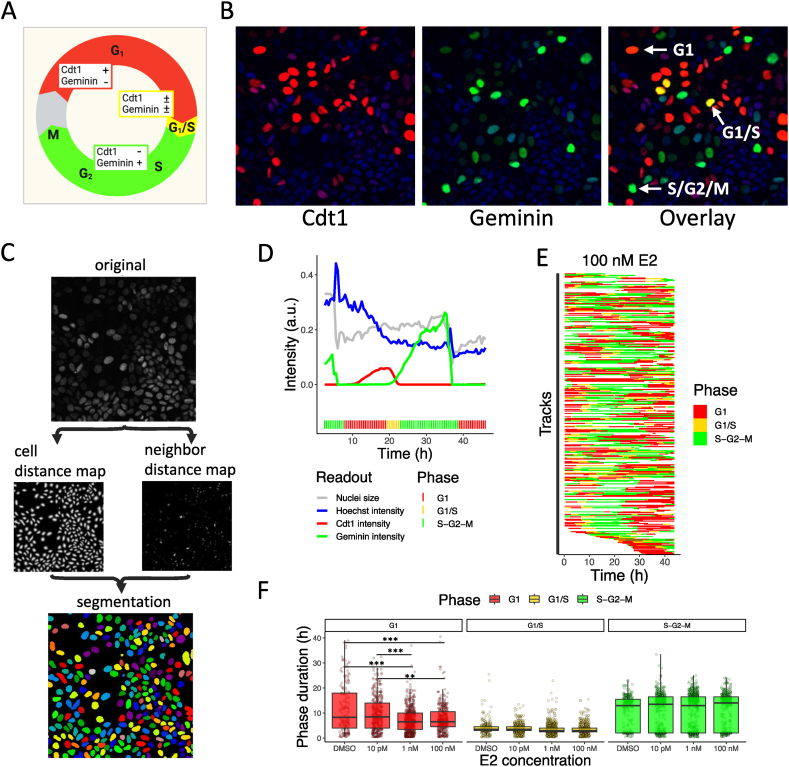
**Live-cell imaging of cell cycle progression in MCF7-FUCCI cells.** A) Fluorescent cell colors adopted during cycling. Cells are red in G1 phase when Cdt1 is present and green during S, G2 and M phase when only Geminin is expressed. During G1/S transition both Cdt1 and Geminin are expressed, and cells display a yellow color. Cells are colorless (indicated with grey color) immediately after mitosis. B) Example images of MCF7-FUCCI cells with Cdt1 in red (left panel), Geminin in green (middle panel) and their overlay, which reveals the yellow color in cells that express both Cdt1 and Geminin (right panel). C) Example images of nuclei segmentation results with the original image, cell distance and neighbor distance predictions, and the segmentation result. D) Example track of a cell with the Cdt1, Geminin and Hoechst intensity and nucleus size over time. The colored bar at the bottom displays the assigned cell cycle phase per timepoint. E) Assigned cell cycle phases for cells after 100 nM E2 exposure. F) Distribution of cell cycle phase lengths at different E2 exposure conditions. Data from one experiment. ∗p < 0.01; ∗∗p < 0.001, ∗∗∗p < 0.0001.

To examine whether our model predictions were consistent with cell cycle progression in living cells, we exploited the cell cycle data published in Ref. [51] by quantifying cell cycle phase durations of individual MCF7-FUCCI reporter cells. FUCCI cells have a red or green fluorescent protein bound to respectively Cdt1 and Geminin. Because Cdt1 is expressed during G1 phase and Geminin during S-G2-M phases, the color of a cell indicates its cell cycle phase, i.e., red during G1, yellow during G1/S transition due to the presence of both Cdt1 and Geminin, and green during S-G2-M (Fig. 4A). We used the raw live-cell confocal microscopy data of MCF7-FUCCI reporter cells imaged over 48 h in Ref. [51] (Fig. 4B) to extract cell cycle phase durations. The high resolution in time and space allowed us to reliably identify single cells with a convolutional neural network (CNN)-based segmentation method. Specifically, two CNNs independently predicted distance and neighbor distance maps on the basis of nuclear Hoechst intensities, which jointly led to excellent predictions of segmented nuclei (Fig. 4C).

Based on these segmentations, we generated single cell tracks and simultaneously measured Cdt1 and Geminin fluorescence intensities in those cells (Fig. 4D). These data allowed us to determine per cell and at every timepoint the cell cycle phase (Fig. 4E), and consequently the duration of the cell cycle phases after different E2 exposure conditions (Fig. 4F). After exposure to E2, the duration of the G1 phase was slightly but significantly shortened compared to control conditions without E2 (median without E2: 8.25 h; median with 100 nM E2: 6.50 h), which was consistent with our cell cycle simulations (Fig. 3G–H). Comparing the single cell tracks in DMSO (Supplementary Fig. 5A) and after 100 nM E2 exposure (Fig. 4E) clearly indicated longer G1 phases for cells in control conditions, especially after the first cell division. Consistently, the shorter G1 phase after E2 exposure coincided with a faster increase in cell counts in presence than in absence of E2 (Supplementary Fig. 5B). As expected based on the time required for E2 signaling and cell division, this E2-mediated acceleration only started to occur after an initial lag period of 30–40 h. Overall, phases were slightly longer in our simulations compared to the experimental data. Nevertheless, the qualitative agreement between the model simulations and our model predictions suggested that our model correctly captures the effect of E2, GREB1 and PR on cell cycle phase durations.

### 2.4 *In silico* knockdown successfully predicts cell cycle phase durations

**Fig. 5:**
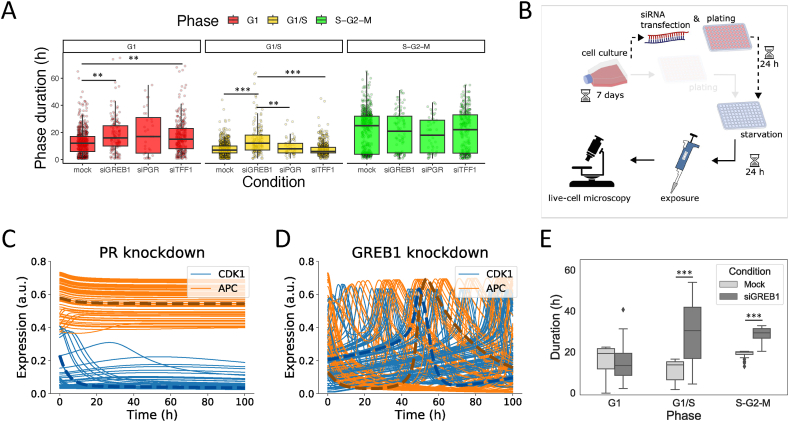
**Measured and predicted cell phase durations after protein knockdowns.** A) Distribution of cell cycle phase lengths without (mock) or after knockdown of GREB1, PR and TFF1 proteins and with exposure to 100 nM E2. Data combined from two biological replicates. B) Flow of subsequent experimental phases used to reproduce the knockdown experiment *in silico*. C-D) Simulations of CDK1 and APC activities in 100 unsynchronized cells after (C) PR knockdown or (D) GREB1 knockdown during the first 100 h after 100 nM E2 exposure. Typical time traces have been highlighted for clarity (dashed lines; dark orange, APC; dark blue, CDK1). E) Quantification of *in silico* cell phase durations for the trajectories in D. ∗p < 0.01; ∗∗p < 0.001, ∗∗∗p < 0.0001.

To further test the validity of our model, we questioned whether our model could predict the effect of GREB1 and PR knockdown on cell cycle progression. Similar to the FUCCI data under different E2 exposure conditions, we created single cell tracks for the FUCCI siRNA transfection data published in Ref. [51] and quantified phase durations to compare cell cycle progression in mock condition, i.e., without protein knockdowns, or after protein knockdown (Fig. 5A). Knockdown of GREB1 increased the duration of the G1 and G1/S phase, and TFF1 knockdown increased G1 phase duration. After PR knockdown the spread in G1 phase durations became high and a small number of quantifiable tracks remained. Visual comparison of the single cell tracks showed that G1 phases were strongly increased after PR knockdown compared to the mock condition (Supplementary Figs. 6A–B). Similarly, the differences in G1 phase duration after TFF1 knockdown and G1/S phase duration after GREB1 knockdown were most apparent in the overviews of single cell tracks (Supplementary Figs. 6C–D), presumably because in the overall statistics we only included measurements for which both phase initiation and termination were visible in the imaging period. These results demonstrate the regulatory role of ERα as well as its downstream targets GREB1, PR and TFF1 on MCF7 cell cycle progression.

To compare the experimental results to model predictions, we mimicked the knockdown experiment *in silico* by introducing a complete knockdown of PR or GREB1 24 h prior to the 24 h starvation period (Fig. 5B). Simulations of this model predicted that the oscillations disappeared completely after PR knockdown (Fig. 5C). Obviously, phase durations cannot be determined in absence of oscillations, but the sustained low expression of CDK1 indicated that *in silico* cells were residing in G1 phase, which was consistent with experimental observations (Supplementary Fig. 6C). Like for PR knockdown, oscillations were much slower for GREB1 knockdown compared to mock conditions (Fig. 5D), which was apparent from the quantified simulated cell trajectories (Fig. 5E). Specifically, GREB1 knockdown caused a considerable increase in G1/S duration, similar to the effect observed in the experimental data. However, the S-G2-M phase duration was also slightly increased in our simulations, which was not reflected by the data. In conclusion, with the combined E2 signaling and cell cycle model, we were able to explain the most important findings in the experimental data, i.e., G1 arrest after PR knockdown and a prolonged G1/S phase transition after GREB1 knockdown. Therefore, our model provides valuable insight in the regulation of cell cycle progression by E2-regulated proteins.

## 3 Discussion

EDCs interfere with endogenous hormone regulation and can cause, among others, disturbance of developmental and reproductive processes [66] and immune system functioning [67], depending on the targets they bind to. Estrogenic EDCs can alter cell cycle progression, which is associated with an increased risk of developing cancer [68,69]. Therefore, it is important to obtain a detailed understanding of how EDCs affect the ERα signaling pathway and what the quantitative influence of this signaling is on cell cycle progression. This knowledge will greatly help our subsequent understanding of the impact of estrogenic EDCs.

In this study, we used protein expression data of high time resolution after exposure to different E2 concentrations and ERα target knockdowns GREB1, PR and TFF1 to build an ODE model that can both describe the upregulation of ERα targets under influence of E2, and predict the effect of knockdown of single proteins on the expression of the others. In our model, binding of both GREB1 and PR to the E2-ERα complex was necessary to correctly describe the protein dynamics after E2 exposure and protein knockdowns, supporting prior evidence on the transcriptional ERα/PR complex formed in response to E2 exposure [27] that drives cell cycle progression.

In addition, we studied the effect of activation of the E2 signaling pathway on cell cycle progression by creating single cell tracks of MCF7-FUCCI cells and quantifying phase durations. GREB1, PR and TFF1 affected cell cycle phase duration in a distinct manner. We integrated our E2 signaling dynamics model with an elementary cell cycle progression model to understand the relation between subcellular processes and cell behavior. Due to the simplicity of the adopted cell cycle model with APC and CDK1 as the only species, and the lack of direct interactions of GREB1, PR and ERα with APC and CDK1, the coupling of the two models took into account those indirect interactions. Theoretically, CDK1 activity is stimulated by GREB1, through the GREB1-dependent activation of Akt [45] and subsequent inactivation of p21 [[63], [64], [65]]. Similarly, E2 and PR stimulate the production of cyclin D1 [[35], [37], [44], [70]], which is expected to accelerate the transition from G1 to S-G2-M, which we modeled by making the inactivation of APC dependent on the availability of the E2-ERα complex and PR. Experimental support for these interactions would further justify the chosen approach.

Mimicking knockdown experiments with the coupled models, we found that knockdown of GREB1 caused a prolonged G1/S phase transition due to prolonged CDK1 activation in absence of GREB1. In contrast, our model suggested that slow APC inactivation after PR knockdown explains the resulting G1 arrest. Although the prolonged G1 phase after TFF1 knockdown corroborates previous reports [48,51], we did not include TFF1 in the model due to unreliable predictions at timepoints much later than 72 h. Therefore, further examination of the regulation of TFF1, particularly its degradation, is necessary to accurately describe its long-term dynamics.

In general, our study shows that combining a signaling pathway model with a molecular cell cycle model can be a successful strategy to explain the effect of protein dynamics on downstream cell fate. Nevertheless, our approach is only one of many options to integrate intracellular dynamics with cell cycle progression. Indeed, the dynamics of proteins involved in cell cycle progression have been modeled extensively [55], in different gradations of complexity. For example, there are simple models that consist of merely two to four proteins [57,58,71,72], and complex models that contain more than 20 state variables [59,73,74]. Models that focus on cell cycle phase transitions often explicitly model the cell cycle phases rather than intracellular protein dynamics [56,60,75,76]. Although these models are well suited to compare simulations with FUCCI cell cycle progression data, it is challenging to integrate these with a model describing signaling components that affect the cell cycle, such as the E2 signaling pathway. Alternatively, models that do describe protein signaling dynamics involved in multiple cell cycle phases [77,78] could be used for this purpose, but are complicated to properly parameterize in absence of detailed time-course measurements of cell cycle protein measurements for the MCF7 cells we utilized in our experiments. Therefore, as proof of principle we asked whether a simple cell cycle model would be sufficient to qualitatively connect our E2 signaling model to the cell cycle. Specifically, we elected the elementary cell cycle model of Bae et al. (2019) [58], which offered a rough approach to classify cell cycle phases based on the dynamics of CDK1 and APC activities (using arbitrary thresholds for these activities to determine the cell cycle in which a cell resides). Because we only studied the effect of GREB1 and PR on cell cycle progression and these proteins play distinct roles in cell cycle progression, this simple model gave qualitatively adequate results. However, future work should focus on detailed quantitative predictions of cell cycle durations as influenced by E2 signaling, which will require a more complex cell cycle model which includes for example bistable switches for mitotic entry [[79], [80], [81], [82]], and the same may be true for incorporation of the effects of other proteins with additional and potentially subtle effects on the cell cycle.

We showed that predictions of our combined E2 signaling and cell cycle model qualitatively matched the knockdown effects on cell cycle phase durations observed in our experimental data. However, the overall duration of cell phases was considerably higher in our simulations than in the experimental data, which at first sight is surprising. To generate our simulations, we set the *k*_*GREB1*_ and *r* parameters such that the expected value for the mean division time was approximately 48 h, similar to the proliferation rate at population level in the data (Supplementary Fig. 4B). Moreover, we selected cell cycle model parameters such that the length of the G1 and S-G2-M phases were comparable, as was the case in our experimental data and in data reported elsewhere [60,83]. The quantitative mismatch in phase durations between experiment and model is likely partially due to the limited time window of observation in imaging experiments. In many tracks, the total duration of at least one cell cycle phase could therefore not be determined. Measurements of cells with long phases could therefore often not be taken along in the summary statistics, which caused an overrepresentation of cells with short phases in our data.

An additional mismatch between observed and *in silico* cell cycle phases was the lower variability in phase durations in simulated cells compared to MCF7 cells. Clearly, the variation in phase durations between cells is not only caused by the phase cells are in at time of treatment, but also by stochastic variation between cells [60,84,85] which we did not incorporate in our model. Thus, the quantitative discrepancies in cell phase duration could be due to a combination of shortcomings of the data and simulation setup. Various approaches can contribute to solving such problems, e.g., longer measurement time windows, mathematical approaches to correct for censored data [86], and inclusion of stochastic intercellular variation in model parameters.

To correctly capture the protein dynamics of GREB1, PR and TFF1 and their interactions, we found that a physical association between the E2-ERα complex and both PR and GREB1 was required, in addition to E2-ERα and PR-dependent stimulation of TFF1. In this way, the model ensured the interdependency of GREB1 and PR, and GREB1-independency of TFF1. Two prior studies revealed the potential of PR to bind to ERα in the presence of progestins [21,22] and another study showed that ERα/PR-B association as part of a larger complex with the ERα coactivator proline-, glutamate- and leucine-rich protein 1 (PELP1) can also occur when PR is unliganded [27]. Because the media used in our study did not contain progestins, nor was progesterone used as exposure condition, our work corroborates binding of unliganded PR with ERα. However, no study explicitly reported on ERα/PR/GREB1 complex formation, so further research into the physical interactions between these proteins is warranted. This will help to resolve the role of PR, the order of complex formation and the contribution of the various complexes in transcriptional activation of the target genes.

With our approach, we managed to build a model that connects two levels of biological complexity, i.e., E2 signaling dynamics and cell cycle progression. Our model allows quantification of the effect of perturbations in the E2 signaling pathway on cell cycle progression and can support mechanistic understanding of cell cycle regulation by estrogenic EDCs. Further extensions that include the interactions of other proteins or signaling pathways will establish a holistic model for the cellular response to endocrine disrupting chemicals on cell cycle progression. In addition, incorporation of these ODE models in qAOPs involving ERα activation as key event could determine biological tipping points and predict consequent adverse outcomes [61,62,87,88]. For example, AOP 200 in the AOP-Wiki describes the sequence of key events between initial ERα activation and ultimately breast cancer as adverse outcome (see https://aopwiki.org/aops/200). Here, increased proliferation of breast epithelial cells in response to e.g. EDCs is one important key event, yet quantification of this AOP is currently lacking. Our model provides a solid basis to further build this quantitative understanding, supported by additional data on how much various EDCs trigger ERα downstream events. Specifically, in combination with short term *in vitro* data of the BAC-GFP reporters exposed to estrogenic EDCs such as the industrial phenolic bisphenol A or the phytoestrogen genistein, our ODE model can be exploited to predict their effect on proliferation at later timepoints. Moreover, it is important to test the validity of these predictions by also generating cell proliferation data, and if needed the models can subsequently be updated to remedy mismatches. Such next-generation risk assessment approaches are becoming increasingly relevant, as they will reduce time and resources in the safety evaluation of chemicals.

## 4 Methods

### 4.1 Cell culture and exposure

We maintained MCF7 GREB1-GFP, MCF7 PR-GFP, MCF7 TFF1-GFP and MCF7 FUCCI cells (established as described in Duijndam et al., 2021 ^51^) in RPMI-1640 medium modified with l-glutamine, HEPES and phenol red (#22400089, Gibco, ThermoFisher Scientific) and supplemented with 10 % fetal bovine serum (FBS) (#10270106, Gibco, ThermoFisher Scientific), 25 U/ml penicillin and 25 μg/ml streptomycin (#15070063, Gibco, ThermoFisher Scientific) (further referred to as complete medium) at 37 °C under 5 % CO2 atmosphere. The starvation medium consisted of phenol red-free RPMI1640 medium modified with l-glutamine (#11835105, Gibco, ThermoFisher Scientific) supplemented with 5 % charcoal/dextran-treated fetal bovine serum (cdFBS) (#SH30068.03, HyClone, GE Healthcare). We treated cells with 17β-estradiol (E2) (no. E1024, Sigma-Aldrich), as described below. We freshly prepared serial dilutions in DMSO (VWR International) per independent run and further diluted it in starvation medium with a maximum concentration of 0.1 % (v/v) DMSO.

### 4.2 Live-cell imaging

To prepare the GFP reporter cells for 72 h live imaging, we seeded cells in a 384-well black screenstar imaging plate (no. 781866, Greiner Bio-One) in complete medium and after 16–24 h the medium was replaced by starvation medium. Following a 24-h starvation period, and 2 h prior to exposure to E2, we loaded the cells with 100 ng/ml Hoechst 33342 (ThermoFisher Scientific) to visualize the nuclei and removed the Hoechst-containing medium before exposure. We exposed the cells to E2 in a broad concentration range (100 fM to 10 μM E2, with 10-fold dilution steps), and captured images every hour for 72 h (2 positions per well, 2 wells per condition). We performed the experiment in triplicate, i.e., with a new batch of cells, to generate three biologically independent replicates.

We used the siRNA transfection data and FUCCI imaging experiments published by Duijndam et al., 2021 [51]. In short, in these experiments, cells were reversely transfected with siRNA in a 96-well plate. 16–24 h after transfection, the medium was replaced by starvation medium. After a 24-h starvation period, and 2 h prior to exposure to E2, cells were loaded with Hoechst, and Hoechst-containing medium was removed before exposure. Cells were exposed to E2, and images were captured every hour for 24 h (GFP reporters, 4 positions per well, 2 wells per conditions, 3 biological replicates) or every half hour for 48 h (FUCCI reporter, 4 positions per well, 2 wells per condition, 1 biological replicate for the experiment with different E2 concentrations and 3 biological replicates for siRNA-mediated knockdown experiments.

To evaluate the response to potential estrogenic stimuli within the complete medium utilized in our experimental set-up and estimate the rate of decay of these stimuli during starvation, we conducted a starvation experiment with the GFP reporter cells under one of the following conditions: (I) cells were maintained in complete medium prior to plating and subsequently plated in complete medium, and the complete medium was replaced by starvation medium two days later, or (II) cells were maintained in complete medium prior to plating and subsequently plated in complete medium, and the complete medium was replaced by starvation medium the next day, or (III), cells were maintained in complete medium prior to plating and subsequently plated in starvation medium, or (IV) cells were maintained in starvation medium prior to plating and subsequently plated in starvation medium (Supplementary Fig. 3A). We seeded cells in a 384-well black screenstar imaging plate (no. 781866, Greiner Bio-One). In each condition, we loaded cells with Hoechst 48 h after plating and 2 h prior to imaging, and the Hoechst-containing medium was removed immediately before imaging (as described above). We captured images at 0, 24 and 48 h (2 positions per well, 2 wells per condition, 1 biological replicate). Hoechst, GFP and RFP levels were monitored using a Nikon TiE2000 confocal laser microscope (lasers: 408, 488, 561, 647 nm), equipped with an automated stage, perfect focus system, and climate chamber (at 37 °C under 5 % CO2 atmosphere). Imaging was performed with a Nikon Plan Apo 20 × magnification objective lens (1 × or 2 × optical zoom) using NIS elements software (Nikon).

### 4.3 Population level image analysis

To obtain population level protein expression data matrices from the 72 h BAC-GFP live-cell imaging data, we used CellProfiler version 2.1.1. First, individual nuclei were segmented with an in-house developed module for Watershed Masked Clustering (WMC) [89]. The resulting binary image was used for nuclei identification using the IdentifyPrimaryObjects module, followed by the IdentifySecondaryObjects module with the N-distance method of 8 pixels to determine the cytoplasmic region. To obtain the GFP intensities in the nuclei and cytoplasm, we applied the MeasureObjectIntensity module. After export to HDF5 files, we extracted relevant features from these files, i.e., the integrated cytoplasmic (for GREB1 and TFF1) and nuclear (for PR) intensities and cell count, using the H5CellProfiler method by Wink et al. (2022) [90].

Following this initial image analysis, we performed additional data analysis steps. First, we normalized cell counts by calculating the ratio between the cell count at each timepoint and the number of cells at the first measurement timepoint. Second, in control experiments with exposure of cells to DMSO, the GFP intensities decreased over time. To correct the GFP intensities for this observed decrease in control conditions, we computed the ratio between measurements with E2 exposure and those with DMSO exposure (taking the average of the technical replicates for DMSO) per biological replicate, reporter cell line and timepoint. Third, due to likely erroneous measurements at late timepoints a sudden temporary drop in intensity of GREB1 and TFF1 occurred in two out of three biological replicates. Therefore, we removed the data collected at time points later than 55 h.

### 4.4 Nuclei segmentation for cell tracking

To improve the identification of distinct nuclei and acquire reliable segmentation for single cell tracking of the FUCCI experiments (see section Single cell tracking), we adapted and trained an existing U-Net convolutional neural network [91] established by Scherr et al. (2020) [92]. In this model, a single neural network with two parallel decoder paths predicts both a cell distance map (CDP) and a neighbor distance map (NDP). These maps (construction explained in more detail below) quantify the distance to either pixels belonging to other cells (NDP) or to any pixel belonging to other cells or background (CDP). To avoid competition between the CDP and NDP predictions in the architecture of the original model, we separated the single neural network with two parallel decoder paths into two neural networks each with their own decoder path. The neural networks were built using the Pytorch library in Python version 3.7.10, and are available via https://doi.org/10.5281/zenodo.13763386.

To improve prediction performance of the pre-trained neural network on our images, we created training sets using manual segmentation on 20x magnification images in ImageJ (16 images, with in total 2312 examples of segmented nuclei). Because automated segmentation performs well on high-resolution images and its assistance speeds up the manual segmentation process, we used CellProfiler's IdentifyPrimaryObjects module followed by manual correction for 40x magnification images (20 images, with each approximately 50–100 objects per image). Prior to training, these segmented images were converted to CDPs and NDPs with a pre-processing pipeline in Python attached to the model. Following Scherr et al. (2020) [92], we created the CDP by applying a distance transform to each cell, i.e., each pixel of an object obtains a value for the distance to the nearest pixel not belonging to the same object, followed by normalization of the distance transform to a [0, 1] interval and combining the results per object to create a single CDP. For the creation of an NDP, each single segmented object was removed one at a time from the segmented image by subtraction, which resulted in a set of images that all had one segmented object missing. We subsequently inverted the resulting images and applied a distance transform. This image was masked to the segmented image of the removed cell, normalized to a [0, 1] interval and inverted again. Finally, we combined all the resulting neighbor distance transformed objects, applied grayscale closing with a kernel of 3 by 3 and raised the pixel values to a power of 10 to obtain a steep gradient within objects.

We augmented the training set by mirroring the images, rotating them with 90, 180 and 270° before and after mirroring, and applying an elastic deformation [93] with sigma = 6 and alpha = 40 and subsequently mirroring and rotating the deformed images. During training, the images for training were shuffled and randomly split into a training set containing 90 %, and an evaluation set containing 10 % of the images. During training of the model on the 40x magnification images, we used a batch size of 10 for a maximum of 15 epochs for the NDP model and 10 epochs for the CDP model. We changed the number of epochs to 20 and 5 for training the NDP and CDP models on the 20x magnification images, respectively. To create the final CDP and NDP predicted images to be employed for segmentation, we used seed extraction and a binary mask followed by a watershed on the prediction output images of the model. First, we applied Gaussian smoothing on both the cell and neighbor distance predictions with sigma = 1.5. We used a threshold of 0.15 to the smoothed cell distance prediction to generate a binary mask. Next, the smoothed neighbor distance prediction was squared and subtracted from the smoothed cell distance prediction, which provided the basis for the seed map. We generated the final seeds by applying a threshold of 0.25 to the subtracted image and used the mask and seeds as input for the watershed.

### 4.5 Single cell tracking

We used the U-net-based segmentation predictions of the FUCCI images as direct input for a single-cell tracking pipeline in CellProfiler 4.1.3. Within the TrackObjects module, we applied the overlap method to create single-cell tracks, allowing for a maximum distance of 40 pixels between the objects' centers of mass at subsequent timepoints. Because CellProfiler uses the same object ID for all cells within one lineage, i.e., the parental cell and all its descendants, we renamed the tracks of sequential daughter cells with the CPtrackR package in R (available at https://doi.org/10.5281/zenodo.4725472) to obtain unique track IDs for each track. For example, instead of ID 1 for both a mother cell and her two daughter cells, the mother cell with ID 1 produces daughters with IDs 1.1 and 1.2. Equivalently, descendants of daughter 1.1 are assigned ID 1.1.1 and 1.1.2. The resulting tracking data were subsequently filtered and processed in R version 4.2.1 to remove unreliable tracks that originate from mistakes by the automated tracking algorithm. First, we removed track IDs of less than 5 timepoints in length and without descendants from the data set, i.e., objects that appear and rapidly disappear. Second, for every final-generation descendant we created a single ‘family’ track containing the subtracks of all ancestors, such that every row of the data set contained a unique, full track from initial parent up to the last generation descendant. Note that this implies that the ancestor subtracks of these rows are exactly repeated across multiple family tracks. Third, we removed short family tracks of 30 timepoints or less. Fourth, family tracks with more than 4 divisions based on the Geminin-GFP intensity (as defined below) were removed from the data set.

For every segmented nucleus we used the MeasureObjectIntensity module to extract the Geminin-GFP and Cdt1-RFP intensities inside the nuclei. In combination with tracking, this delivered sequences of the readouts, i.e., Cdt1-RFP, Geminin-GFP, Hoechst mean intensities in the nuclei and the nuclear size. We normalized each readout using min-max normalization, i.e., ${\vec{x}}^{\prime }=\frac{x_{i}-\min\left(\vec{x}\right)}{\max\left(\vec{x}\right)-\min\left(\vec{x}\right)}\text{,}$ with *x*_*i*_ the single measurements and *x* all measurements per readout channel within one experiment. After careful examination of the tracks, we deduced that irregularities in the Geminin-GFP intensity were most indicative for unreliable tracks. To filter out uncertain tracks, we determined cell divisions based on the Geminin-GFP expression pattern. For this purpose, we smoothed the Geminin readout with a rolling mean with a window of 2 timepoints and calculated the difference *d* between consecutive expression values. We considered the logical expression

1 celldivision=d<−0.5·SD(Geminin),

with SD the standard deviation, to determine whether a cell division occurred, i.e., a cell divided when its Geminin-GFP value decreased more quickly than a threshold. We removed tracks with more than 4 cell divisions from the data set.

To remove small fluctuations in measurements, we applied a rolling mean with a centered window of 10 timepoints on the raw values of all readouts. Subsequently, we computed and log-transformed the Geminin:Cdt1 ratio according to the conditions

2 $$r=\left\{ \begin{array}{ll} -100, & \left(\text{Geminin}=0\right)\vee \left(\text{Geminin}<0.0001\wedge \text{Cdt}1<0.0001\right)\\ 100, & \left(\text{Cdt}1=0\right)\wedge \left(\text{Geminin}>0.0001\right)\\ {\mathrm{Log}}_{10}\left(\frac{\text{Geminin}}{\text{Cdt}1}\right), & \text{otherwise}. \end{array}\right.$$

We then determined the cell cycle phase per track according to the following conditions:

3 $$\text{cell}\;\text{cycle}\;\text{phase}=\left\{ \begin{array}{ll} G1, & \left(r=-100\right)\vee \left(\text{Cdt}1>\text{Geminin}\right)\\ S-G2-M, & \left(r=100\right)\vee \left(\text{Geminin}>\text{Cdt}1\right)\\ G1/S, & \left|r\right|<2 \end{array}\right.\text{.}$$

Despite the filtering steps and application of the rolling mean to prevent small measurement fluctuations leading to repeated cell cycle switching, some irregularities in phase assignment still occurred. We therefore applied an additional correction: if the determined cell cycle phase switched to a different phase but reverted within maximally 4 timepoints, we corrected this by replacing the phase assigned to the timepoints with the alluded temporary phase change with the phase before the switch. In addition, irregularities occurred in cells that went from S-G2-M to G1 phase during mitosis, a transition in which, for a short period of time, the |*r*| value could become smaller than 2, which led to a cell cycle phase assignment of G1/S. In these cases, we corrected the G1/S phase to become G1 (i.e., transition from S-G2-M directly to G1-S was not allowed).

Following the above assignment of cell phases, we quantified the phase durations for each unique track. For this purpose, we removed the parts of family tracks that led to parental track duplicates and very short remaining subtracks of 5 or less timepoints in length (after removal of the parental parts of a track). For statistical analysis, we included only the cell cycle phases for which the start and end were both contained in the measurement time window of 48 h. The non-parametric Welch's one-way ANOVA followed by a Games-Howell pairwise comparison was applied to identify significant differences in phase durations between conditions, i.e., E2 concentrations and siRNA knockdown conditions.

### 4.6 E2 signaling models

We created three ODE model variants to simulate the E2 signaling pathway and to predict the inter-protein dependencies revealed by the knockdown experiments (available via https://doi.org/10.5281/zenodo.13763386). Apart from mass-action kinetics to describe synthesis and degradation rates, we modeled binding, modification and stimulation of state variables with terms of the form $p\cdot \frac{X\;\cdot \;Y}{1+X+Y}\text{,}$ with *p* a parameter and *X* and *Y* state variables, to prevent unlimited accumulation of these entities in the system. In Model I, E2 (*E2*) binds to ERα (*ER*) to form an E2-ERα (*E2_ER*) complex with binding rate *b*_*E2_ER*_. In contrast to E2, which is only depleted through E2-ERα complex formation and subsequent degradation of this complex with rate *d*_*E2_ER*_, ERα is replenished by basal synthesis (*s*_*ER*_) and degraded with rate *d*_*ER*_. GREB1 (*GREB1*) associates with the E2-ERα complex at rate *b*_*E2_ER_GREB1*_ to form an E2-ERα/GREB1 (*E2_ER_GREB1*) complex. The differential equations for E2, ERα and their complexes thus become:

4 $$\frac{\text{dE}2}{\text{dt}}=-\frac{{\;b}_{E2\_\text{ER}}\;\cdot \;E2\;\cdot \;\text{ER}}{1+E2+\text{ER}}\text{,}$$

5 $$\frac{\text{dER}}{\text{dt}}=s_{\text{ER}}-\frac{b_{E2\_\text{ER}}\;\cdot \;E2\;\cdot \;\text{ER}}{1+E2+\text{ER}}-d_{\text{ER}}\cdot \text{ER,}$$

6 $$\frac{\text{dE}2\_\text{ER}}{\text{dt}}=\frac{b_{E2\_\text{ER}}\;\cdot \;E2\;\cdot \;\text{ER}}{1+E2+\text{ER}}-\frac{b_{E2\_\text{ER}\_\text{GREB}1}\;\cdot \;E2\_\text{ER}\;\cdot \;\text{GREB}1}{1+E2\_\text{ER}+\text{GREB}1}-d_{E2\_\text{ER}}\cdot E2\_\text{ER},\text{and}$$

7 $$\frac{\text{dE}2\_\text{ER}\_\text{GREB}1}{\text{dt}}=\frac{b_{E2\_\text{ER}\_\text{GREB}1}\;\cdot \;E2\_\text{ER}\;\cdot \;\text{GREB}1}{1+E2\_\text{ER}+\text{GREB}1}-d_{E2\_\text{ER}\_\text{GREB}1}\cdot E2\_\text{ER}\_\text{GREB}1\;\text{.}$$

Apart from basal synthesis of proteins GREB1, PR and TFF1 (*TFF1*) with rate constants *s*_*GREB1*_, *s*_*PR*_, *s*_*TFF1*_, their degradation with rate constants *d*_*GREB1*_, *d*_*PR*_, *d*_*TFF1*_, and consumption of GREB1 into the E2-ERα/GREB1 complex, the synthesis of the three proteins is stimulated by the E2-ERα/GREB1 complex. Specifically, the formation of GREB1, PR and TFF1 is stimulated by the E2-ERα/GREB1 complex with rate *stim*_*GREB1*_*, stim*_*PR*_, and *stim*_*TFF1*_, respectively. The full ODEs for GREB1, PR and TFF1 thus become:

8 $$\frac{\text{dGREB}1}{\text{dt}}=s_{\text{GREB}1}+\frac{{\text{stim}}_{\text{GREB}1}\;\cdot \;E2\_\text{ER}\_\text{GREB}1}{1+E2\_\text{ER}\_\text{GREB}1}-d_{\text{GREB}1}\cdot \text{GREB}1\text{,}$$

9 $$\frac{\text{dPR}}{\text{dt}}=s_{\mathrm{PR}}+\frac{{\text{stim}}_{\mathrm{PR}}\;\cdot \;E2\_\text{ER}\_\text{GREB}1}{1+E2\_\text{ER}\_\text{GREB}1}-d_{\mathrm{PR}}\cdot \mathrm{PR},\text{and}$$

10 $$\frac{\text{dTFF}1}{\text{dt}}=s_{\text{TFF}1}+\frac{{\text{stim}}_{\text{TFF}1}\;\cdot \;E2\_\text{ER}\_\text{GREB}1}{1+E2\_\text{ER}\_\text{GREB}1}-d_{\text{TFF}1}\cdot \text{TFF}\;\text{.}$$

For Model II, we adjusted Equations 8, 10) to make GREB1 and TFF1 synthesis in addition dependent on PR (besides dependence on the E2-ERα/GREB1 complex). In addition, we made TFF1 production independent of GREB1. Thus, in this model TFF1 was stimulated by the E2-ERα complex and PR in a co-dependent (multiplicative) manner. The new ODEs for GREB1 and TFF1 became:

11 $$\frac{\text{dGREB}1}{\text{dt}}=s_{\text{GREB}1}+\frac{{\text{stim}}_{\text{GREB}1}\;\cdot \;E2\_\text{ER}\_\text{GREB}1\;\cdot \;\mathrm{PR}}{1+E2\_\text{ER}\_\text{GREB}1+\mathrm{PR}}-d_{\text{GREB}1}\cdot \text{GREB}1,\text{and}$$

12 $$\frac{\text{dTFF}1}{\text{dt}}=s_{\text{TFF}1}+\frac{{\text{stim}}_{\text{TFF}1}\;\cdot \;E2\_\text{ER}\;\cdot \;\mathrm{PR}}{1+E2\_\text{ER}+\mathrm{PR}}-d_{\text{TFF}1}\cdot \text{TFF}1\;\text{.}$$

In Model III, we adjusted the equations once more by creating an intermediate E2-ERα/PR complex (*E2_ER_PR*) prior to GREB1 binding. The complex with GREB1 bound to E2-ERα/PR (*E2_ER_PR_GREB1*) was considered to stimulate the production of GREB1 and PR. Formation of the E2-ERα complex itself (Equations 4, 5, 6)) remained the same, and the ODEs for formation of E2-ERα/PR, E2-ERα/PR/GREB1 and GREB1, PR and TFF1 then became:

13 $$\frac{\text{dE}2\_\text{ER}\_\mathrm{PR}}{\text{dt}}=\frac{b_{E2\_\text{ER}\_\mathrm{PR}}\;\cdot \;E2\_\text{ER}\;\cdot \;\mathrm{PR}}{1+E2\_\text{ER}+\mathrm{PR}}-{\frac{b_{E2\_\text{ER}\_\mathrm{PR}\_\text{GREB}1}\;\cdot \;E2\_\text{ER}\_\mathrm{PR}\;\cdot \;\text{GREB}1}{1+E2\_\text{ER}\_\mathrm{PR}+\text{GREB}1}-d}_{E2\_\text{ER}\_\mathrm{PR}}\cdot E2\_\text{ER}\_\mathrm{PR}\text{,}$$

14 $$\frac{\text{dE}2\_\text{ER}\_\mathrm{PR}\_\text{GREB}1}{\text{dt}}=\frac{b_{E2\_\text{ER}\_\mathrm{PR}\_\text{GREB}1}\;\cdot \;E2\_\text{ER}\_\mathrm{PR}\;\cdot \;\text{GREB}1}{1+E2\_\text{ER}\_\mathrm{PR}+\text{GREB}1}-d_{E2\_\text{ER}\_\mathrm{PR}\_\text{GREB}1}\cdot E2\_\text{ER}\_\mathrm{PR}\_\text{GREB}1\text{,}$$

15 $$\frac{\text{dGREB}1}{\text{dt}}=s_{\text{GREB}1}+\frac{{\text{stim}}_{\text{GREB}1}\;\cdot \;E2\_\text{ER}\_\mathrm{PR}\_\text{GREB}1}{1+E2\_\text{ER}\_\mathrm{PR}\_\text{GREB}1}-\frac{b_{E2\_\text{ER}\_\mathrm{PR}\_\text{GREB}1}\;\cdot \;E2\_\text{ER}\_\mathrm{PR}\;\cdot \;\text{GREB}1}{1+E2\_\text{ER}\_\mathrm{PR}+\text{GREB}1}-d_{\text{GREB}1}\cdot \text{GREB}1\text{,}$$

16 $$\frac{\text{dPR}}{\text{dt}}=s_{\mathrm{PR}}+\frac{{\text{stim}}_{\mathrm{PR}}\;\cdot \;E2\_\text{ER}\_\mathrm{PR}\_\text{GREB}1}{1+E2\_\text{ER}\_\mathrm{PR}\_\text{GREB}1}-\frac{b_{E2\_\text{ER}\_\mathrm{PR}}\;\cdot \;E2\_\text{ER}\;\cdot \;\mathrm{PR}}{1+E2\_\text{ER}+\mathrm{PR}}-d_{\mathrm{PR}}\cdot \mathrm{PR},\text{and}$$

17 $$\frac{\text{dTFF}1}{\text{dt}}=s_{\text{TFF}1}+\frac{{\text{stim}}_{\text{TFF}1}\;\cdot \;E2\_\text{ER}\;\cdot \;\mathrm{PR}}{1+E2\_\text{ER}+\mathrm{PR}}-d_{\text{TFF}1}\cdot \text{TFF}1\;\text{.}$$

Note that for all protein complexes described in models I, II and III, we considered the complexes to naturally degrade. Although complexes might also disappear by dissociation into separate components, we did not include such processes in our models because the lack of experimental measurements on the protein complexes would preclude identification of values for these parameters.

The parameters in these model variants were optimized with our gradient descent-based optimization method described previously [94]. In brief, we employed sensitivity equations and steady state constraints [95] to find the direction of steepest descent during optimization with the least squares method of the SciPy package in Python version 3.7.3 and used Latin hypercube sampling [96] for efficient sampling of the parameter space during parameter initialization. Because we performed background correction and thereby eliminated any effect of residual E2 in the wells, the system can be considered to start in steady state and deprived of E2. Therefore, the initial states of the three complexes formed during E2-dependent signaling were fixed at 0. Because there were no changes over time in protein dynamics observed in concentrations lower than 0.001 nM and adverse effects were observed for 1000 nM and higher, we fitted to the expression data of the concentrations 0.001, 0.01, 0.1, 1, 10 and 100 nM. The effective concentrations, i.e., the concentrations perceived by the cells, were fitted together with initial states of ERα, GREB1, PR and TFF1, and the model parameters. However, we fixed the lowest effective concentration to 0.001 as a reference for the higher concentrations. All degradation and binding parameters were constrained between 0 and 1. In addition, to force the system to start in steady state, we made the synthesis parameters dependent on the degradation parameters and initial states:

18 $$s_{\text{ER}}=d_{\text{ER}}\cdot {\text{ER}}_{\text{init}}\text{,}$$

19 $$s_{\text{GREB}1}=d_{\text{GREB}1}\cdot {\text{GREB}1}_{\text{init}}\text{,}$$

20 $$s_{\mathrm{PR}}=d_{\mathrm{PR}}\cdot {\mathrm{PR}}_{\text{init}},\text{and}$$

21 $$s_{\text{TFF}1}=d_{\text{TFF}1}\cdot {\text{TFF}1}_{\text{init}}\;\text{.}$$

The parameter values for the three models are available in Supplementary Table 1. To compare the model simulations of PR and GREB1 to their respective data, we used the total amount of PR and GREB1, i.e., their free forms and the amount of these proteins captured in complexes.

### 4.7 Cell cycle model

To connect the protein expression dynamics to cell cycle progression, we modified an existing cell cycle model [57,58]. This model simulates the oscillatory dynamics of active CDK1 (*CDK1*) and active APC (*APC*), which are denoted as fractions of total CDK1 and APC. The active forms of these proteins are used to identify the duration of the G1 phase and total duration of the combined S, G2 and M phases. CDK1 and APC are activated with rates *a1* (dependent on cyclin binding) and *a2* (dependent on the fraction of inactive APC (i.e., 1−APC) and on active CDK1), and deactivated with rates *b1* and *b2*, respectively. Active APC inhibits CDK1 through a multi-step process described by a Hill function (dependent on parameters *n1* and *K1*), whereas activation of APC is influenced by active CDK1 via Hill function parameters *n2* and *K2*. In addition, CDK1 has an auto-stimulatory feedback (via Cdc25, which is activated by CDK1 and in turn activates CDK1) modeled with Hill function parameters *n3* and *K3*. In this model, we incorporated the influence of E2-ERα, GREB1 and PR (current biological knowledge on these interactions summarized in introduction and results) to investigate whether we could predict the influence of E2 signaling and protein knockdown on cell cycle progression. To model the stimulation of G1 progression into S phase, we made the inactivation term for APC dependent on the E2-ERα complex and PR, and modified the *b2* parameter by multiplication with factor *r*. In addition, we modeled the stimulatory effect of GREB1 on CDK1 activity with the addition of a GREB1-dependent activation term with rate *k*_*GREB1*_. The equations of the modified model thus became:

22 $$\frac{\text{dCDK}1}{\text{dt}}=a1-b1\;\cdot \;\text{CDK}1\;\cdot \;\frac{{\text{APC}}^{n1}}{{K1}^{n1}+{\text{APC}}^{n1}}+a3\;\left(1-\text{CDK}1\right)\;\cdot \;\frac{\;{\text{CDK}1}^{n3}}{{K3}^{n3}+{\text{CDK}1}^{n3}}+k_{\text{GREB}1}\cdot \text{GREB}1,\text{and}$$

23 $$\frac{\text{dAPC}}{\text{dt}}=a2\;\left(1-\text{APC}\right)\cdot \;\frac{{\text{CDK}1}^{n2}}{{K2}^{n2}+{\text{CDK}1}^{n2}}-r\;\cdot b2\;\cdot \text{APC}\;\cdot \;E2\_\text{ER}\;\cdot \mathrm{PR}\text{.}$$

Depending on the *k*_*GREB1*_ and *r* values, the cell cycle model displayed sustained oscillations under constant E2 availability. Specifically, oscillations disappeared for *k*_*GREB1*_ ≳ 0.0009 (Supplementary Figs. 2A–B) and for r ≲ 0.1 or r ≳ 6.26 (Supplementary Figs. 2C–D). We took the parameter values for the base cell cycle model from Bae et al. (2019), while adjusting the parameters a1, a2, a3, b1 and b2 (dividing them by 11) to obtain an oscillation period of ∼50 h, i.e., close to the actual cell cycle duration of our MCF7 cells. We manually chose the values for the parameters $k_{GREB1}$ and r in the extended model such that the system displayed sustained oscillations for constant E2 availability and the period became ∼48 h. See Supplementary Table 1 for an overview of all parameter values. The cell cycle model is available via https://doi.org/10.5281/zenodo.13763386.

### 4.8 Model simulations

To closely replicate the *in vitro* experimental set-up to obtain cell cycle progression data with our *in silico* model, we distinguished three experimental phases. In the first phase, cells in complete culture medium had sufficient nutrients and growth-stimulating compounds (i.e., compounds functionally equivalent to E2), available to proliferate at a rate of approximately once per 48 h. A starvation phase (phase 2) of 24 h followed phase 1 in which the complete medium was replaced with starvation medium devoid of E2-equivalent compounds to eliminate the growth-enhancing effect of the medium. In phase 3, cells were exposed to starvation medium with the addition of various E2 concentrations, and protein expression dynamics were measured using live-cell imaging (Fig. 3C). In case of a knockdown experiment, there was an additional phase of 24 h between phase 1 and 2, in which the complete culture medium was replaced with complete transfection medium that contained siRNAs (Fig. 5B).

We mimicked these experimental phases with our model. We first simulated a constant low concentration of E2 during culture conditions, to ensure cells were in steady state. Because we could not directly determine the concentration of E2-equivalent compounds in complete medium, we estimated this concentration based on the dynamics of GREB1, PR and TFF1 in complete and starvation medium without addition of E2 (Supplementary Fig. 3B). To achieve this, we first determined the protein degradation rates during starvation (Supplementary Fig. 3C). For this purpose, we used an elementary degradation model for GREB1, PR and TFF1 with equations:

24 $$\frac{\text{dGREB}1}{\text{dt}}={-d}_{\text{GREB}1}\cdot \text{GREB}1\text{,}$$

25 $$\frac{\text{dPR}}{\text{dt}}={-d}_{\mathrm{PR}}\cdot \mathrm{PR},\text{and}$$

26 $$\frac{\text{dTFF}1}{\text{dt}}={-d}_{\text{TFF}1}\cdot \text{TFF}1\;\text{.}$$

We estimated the degradation parameters by fitting this model to the data from the starvation experiment with the modCost and modFit functions of the Flexible Modeling Environment (FME) package in R (parameter values in Supplementary Table 2). Note that these degradation parameter values are lower than the values estimated in the complete model, which could be due to the difference in chemical properties of E2 and the E2-equivalent compounds.

Secondly, we used a simulation of the elementary degradation model to estimate the concentration of growth-stimulating compounds in complete medium before and after 24 h of starvation. Taking the average of the 3 estimates for the differences between 0 and 24 h in starvation, the protein expression in complete medium was 1.5 times higher than after 24 h in starvation medium. Therefore, we assumed that the E2-equivalent concentration in complete medium would also be approximately 1.5 times higher than in starvation medium at the time of exposure.

Third, to obtain an estimate for the E2-equivalent concentration in starvation medium at the time of exposure, we used the unnormalized protein expression data after exposure (Supplementary Fig. 3D). Because the degradation of the proteins in starvation medium was not entirely completed after 24 h, we observed an initial drop in intensity right after exposure, which was subsequently counteracted by exposure to E2. After exposure to 0.01 nM E2, TFF1 protein expression rapidly reached the same level as at timepoint 0, as opposed to GREB1 and PR that had a net decrease in expression even long after timepoint 0 (Supplementary Fig. 3D, second column). In contrast, PR and TFF1 expression levels attained a higher level than at timepoint 0 after 0.1 nM E2 exposure, whereas GREB1 stabilized to approximately the same level as at timepoint 0 (Supplementary Fig. 3D, third column). Therefore, we inferred that the actual E2-equivalent concentration must lie between 0.01 and 0.1 nM and assumed that the E2-equivalent concentration in starvation medium just before exposure would be similar to a nominal E2 concentration of 0.055 nM, i.e., the average of 0.01 and 0.1 nM. Thus, we used 1.5 times the effective concentration at 0.055 nominal concentration as estimate for the E2-equivalent concentration in complete medium, i.e., 0.104 · 1.5 = 0.156, and simulated the model until GREB1, PR and TFF1 protein expressions were in steady state and the active forms of CDK1 and APC displayed sustained oscillations. We took this as the starting point for simulations of phase 1.

To simulate dynamics for 100 individual cells during knockdown, starvation, and exposure, we chose initial states for active CDK1 and active APC at 100 random timepoints within one cell cycle period (Fig. 3F). To replicate knockdown conditions, we removed the positive terms from the ODE equations of the knocked down proteins. We described the 24-h starvation period by fixing E2 at 0. To imitate the exposure phase, we ran simulations with an effective E2 concentration of 0.837, which corresponded to the 100 nM nominal concentration, for a time span of 100 h.

### 4.9 Phase duration quantification *in silico*

We quantified the length of the G1 phase, G1/S transition phase and S-G2-M phase in our simulations per single cell simulation based on the CDK1 and APC activity pattern. Following Bae et al. (2019) [58], we used the minimum of CDK1 activity as transition point from S-G2-M to G1 phase. We adjusted their criterion using the minimum of APC activity as the transition from G1 to S-G2-M, because we needed to calculate the length of the G1/S transition phase. Therefore, we determined a threshold for APC activity, underneath which cells were considered in G1/S transition. Thus, when APC values got below the threshold, cells went from G1 into the G1/S transition phase. Similarly, if APC values rose above the threshold, cells went from G1/S transition phase into S-G2-M phase. The threshold was set at the APC activity minimum plus 5 % of the difference between the first minimum in APC activity and the APC maximal activity that followed this minimum. Note that we used this approach with an (arbitrary) threshold for APC activity to obtain a rough estimate of the expected G1/S transition duration, and to predict how knockdown conditions affect this duration.

## CRediT authorship contribution statement

**M.M. Heldring:** Writing – original draft, Visualization, Software, Methodology, Investigation, Conceptualization. **B. Duijndam:** Writing – original draft, Methodology, Investigation, Conceptualization. **A. Kyriakidou:** Writing – review & editing, Software, Investigation. **O.M. van der Meer:** Writing – review & editing, Software, Investigation. **M. Tedeschi:** Writing – review & editing, Investigation. **J.W. van der Laan:** Writing – review & editing, Supervision. **B. van de Water:** Writing – review & editing, Supervision, Funding acquisition, Conceptualization. **J.B. Beltman:** Writing – review & editing, Supervision, Funding acquisition, Conceptualization.

## Declaration of competing interest

The authors declare the following financial interests/personal relationships which may be considered as potential competing interests: Joost Beltman and Bob van de Water report financial support was provided by European Union. The other authors declare that they have no known competing financial interests or personal relationships that could have appeared to influence the work reported in this paper.
