# Supplementary material for: Interdependency of estradiol-mediated ERα activation and subsequent PR and GREB1 induction to control cell cycle progression
Source: Heliyon. 2024 Sep 24;10(21):e38406. doi: 10.1016/j.heliyon.2024.e38406 (PMC11582769; doi:10.1016/j.heliyon.2024.e38406)
Supplement: Multimedia component 1 [file mmc1.pdf]

Supplementary Data

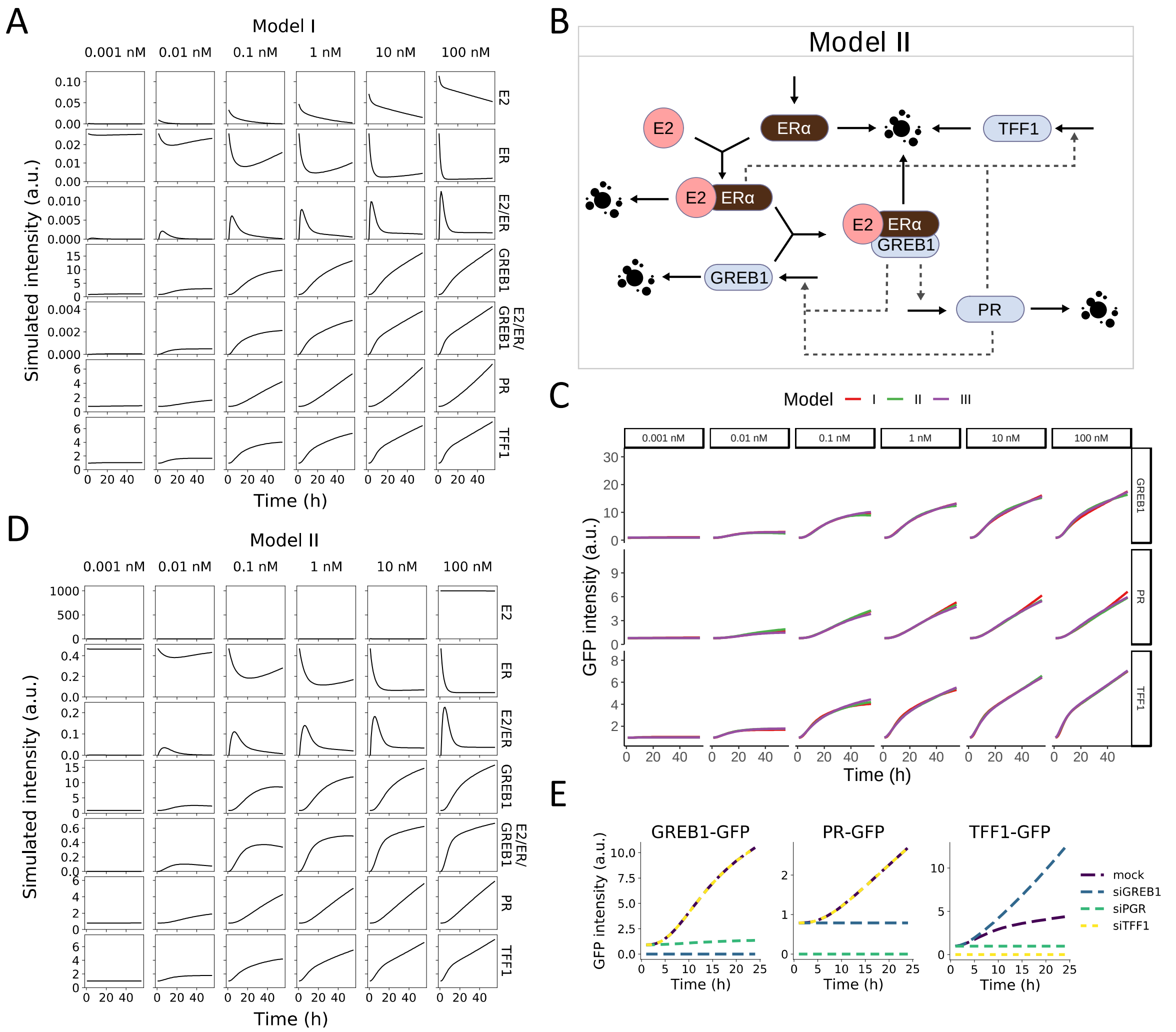

Supplementary Figure 1. Comparison of E2 signaling model variants. A) Inner states for all model variables in Model I. B) Schematic diagram of the E2 signaling model II. E2 and ERα form an E2-ERα complex that stimulates TFF1 in presence of PR and can bind GREB1. E2-ERα/GREB1 complex stimulates synthesis of PR and GREB1, the latter in presence of PR. Solid arrows, synthesis and degradation; dashed arrows, modulation. C) Model simulations for Model I, II and III after parameter calibration (solid lines) to the experimental data (points, three independent replicates separately shown). D) Inner states for all model variables in Model II. E) Predictions of GREB1, PR and TFF1 expression after complete protein knockdown in Model II.

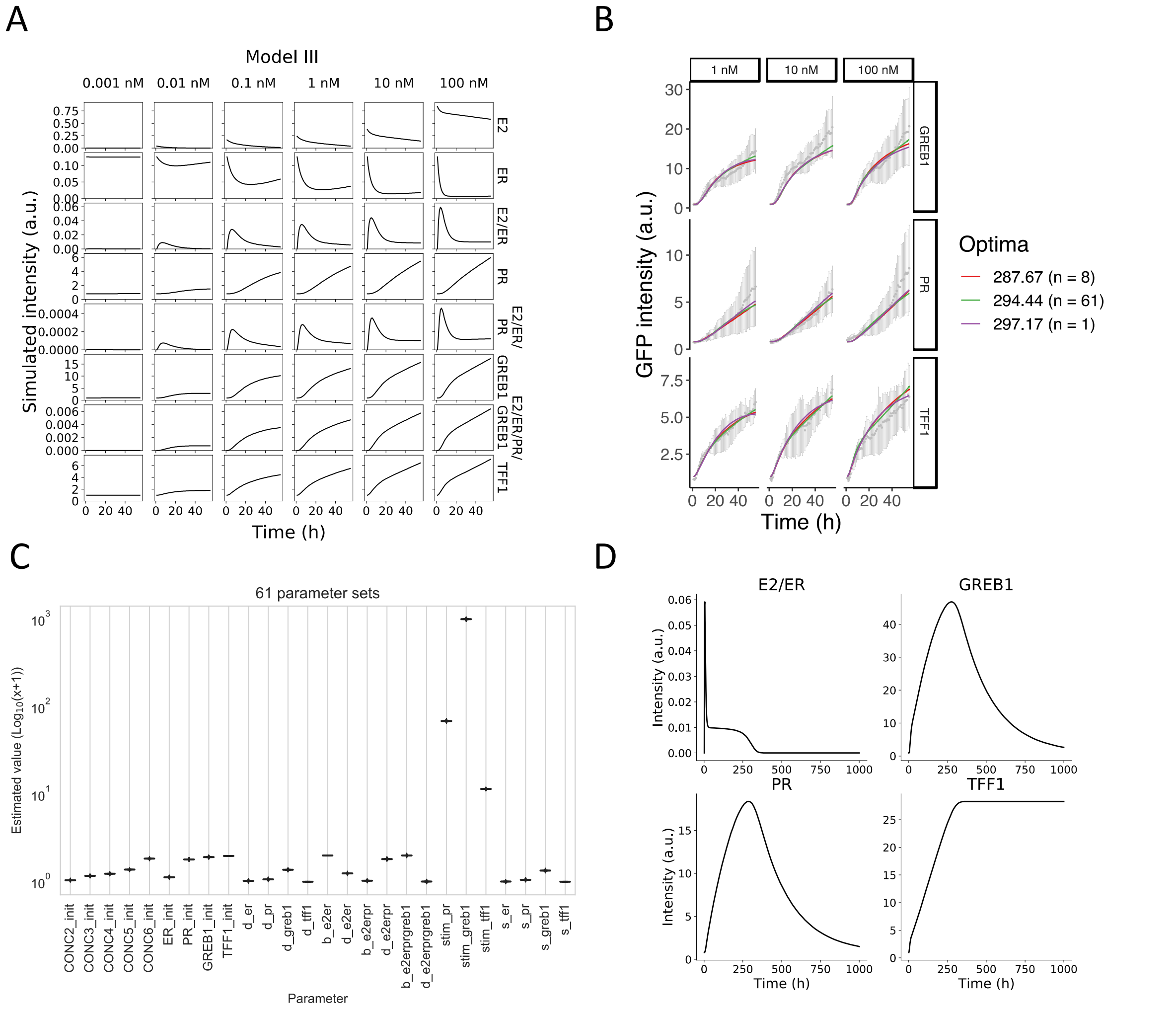

Supplementary Figure 2. Inner states and optimal fits of model III. A) Inner states for all model variables in Model III. B) Comparison of the three distinct optima with lowest cost for the parameter set of model III. C) Boxplot showing the parameter variability between the 61 different fits of model III. D) Simulated protein expression dynamics of model III on a long time scale after 100 nM E2 exposure.

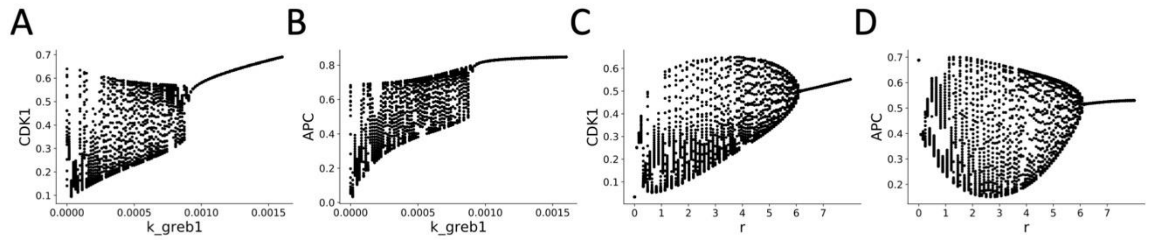

Supplementary Figure 3. Bifurcation plots of CDK1-APC cell cycle model. A-B) Effect of interaction strength parameter *k_greb1* on CDK1 (A) and APC oscillations (B). C-D) Effect of multiplication factor *r* on CDK1 (C) and APC oscillations (D).

A

	Time with respect to the start in starvation medium								
	-72 hr	-48 h	-24 h	0 h	24 h	48 h	72 h	96 h	120 h
I	Culture in complete medium	Plating in complete medium	Complete medium	Replace with starvation medium	Starvation medium	Starvation medium	-	-	-
II	Culture in complete medium	Culture in complete medium	Plating in complete medium	Replace with starvation medium	Starvation medium	Starvation medium	Starvation medium	-	-
III	Culture in complete medium	Culture in complete medium	Culture in complete medium	Plating in starvation medium	Starvation medium	Starvation medium	Starvation medium	Starvation medium	-
IV	Culture in complete medium	Culture in complete medium	Culture in complete medium	Culture in starvation medium	Plating in starvation medium	Starvation medium	Starvation medium	Starvation medium	Starvation medium

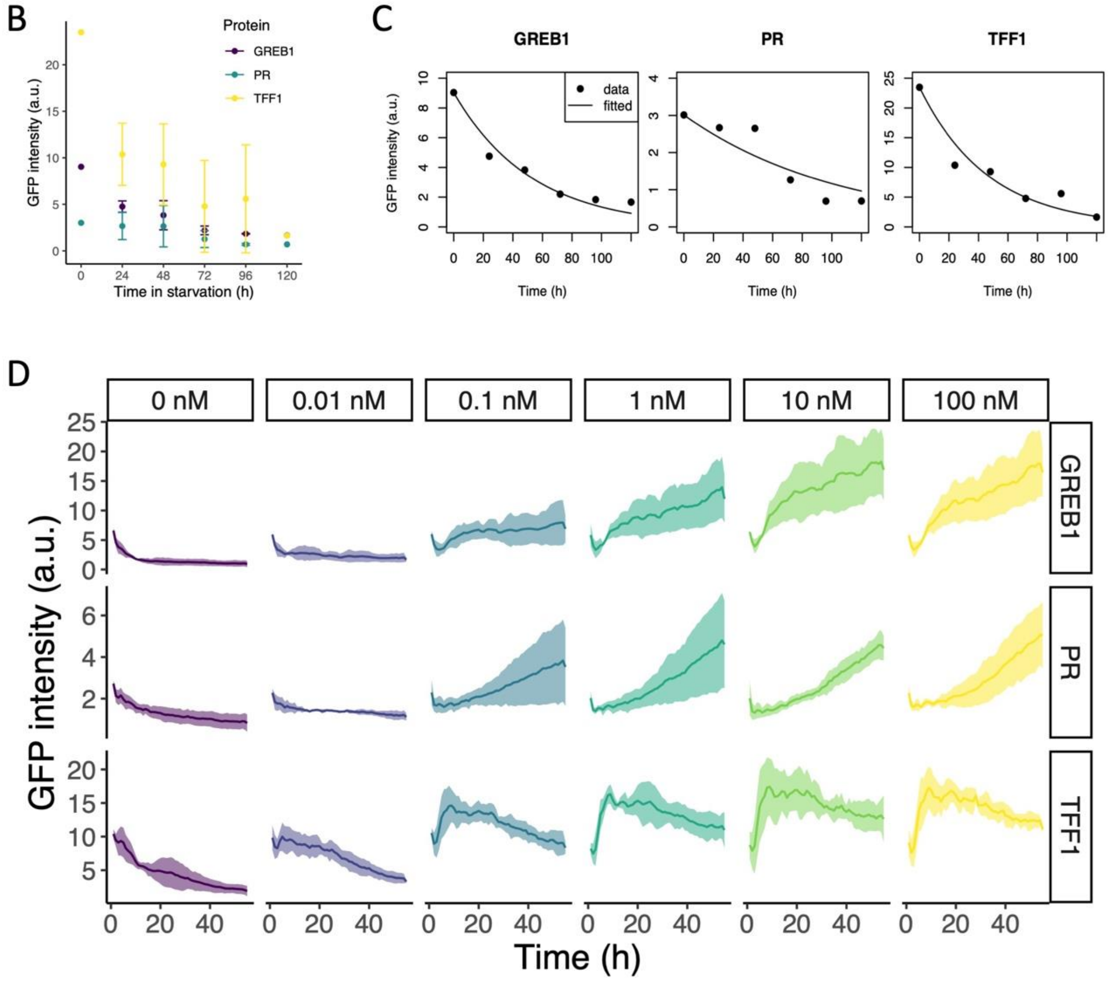

Supplementary Figure 4. Protein expression dynamics in starvation medium before and after E2 exposure. A) Timeline for four experimental conditions to measure protein expression in starvation medium, with the imaging timepoints indicated in grey. B) GREB1, PR and TFF1 protein expression at different timepoints in starvation medium. C) Fits of elementary degradation model to the protein degradation data in starvation medium. D) Unnormalized protein expression dynamics after exposure to different concentrations of E2. Error bars in (B) and (D) represent SD across replicates.

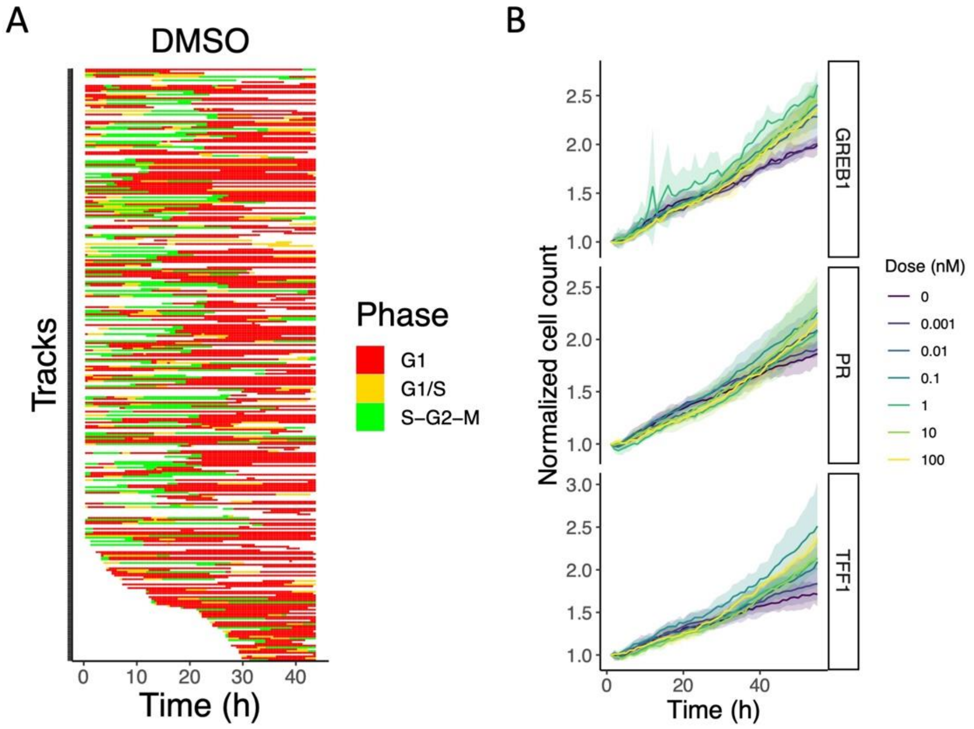

Supplementary Figure 5. Cell cycle progression and population growth in different E2 exposure conditions. A) Assigned cell cycle phases for cells in control DMSO condition, i.e., without E2 exposure. B) Population growth in GREB1-, PR- and TFF1-GFP cell lines at different E2 exposure concentrations. Cell counts are normalized to the values at the first measurement time point.

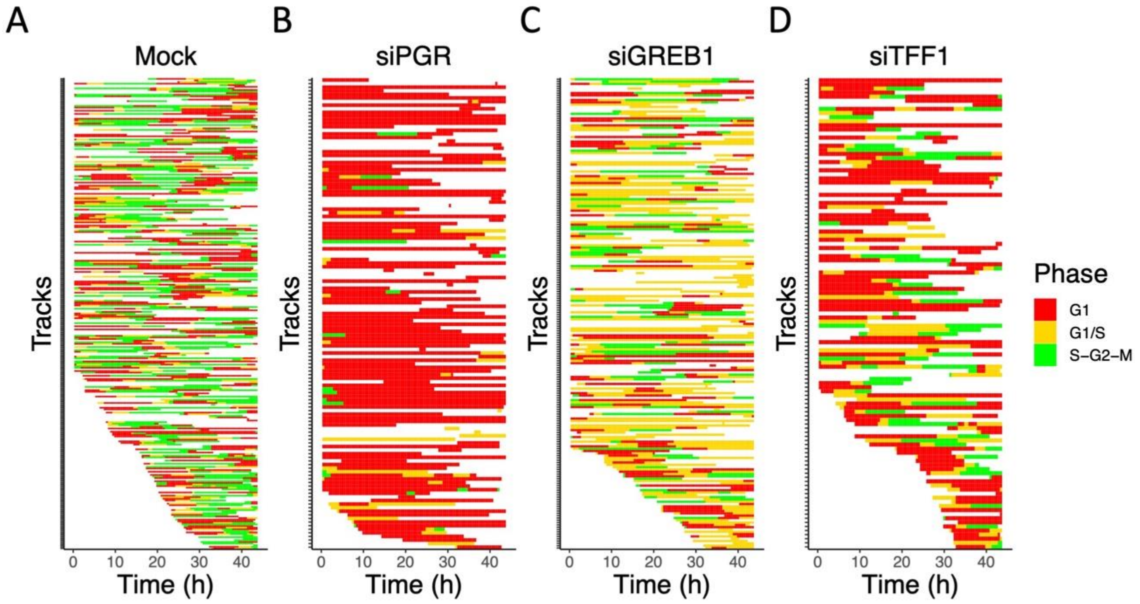

Supplementary Figure 6. Cell cycle phase classification after protein knockdown and exposure to 100 nM E2. A-D) Assigned cell cycle phases for cells in mock condition (A), or after PR (B), GREB1 (C) and TFF1 knockdown (D).

Supplementary Table 1. Values and descriptions of the estimated, fixed and calculated model parameters.

**E2-signaling model parameters**					
**Parameter**	**Value**			**Unit**	**Description***
	**Model I**	**Model II**	**Model III**		
$CONC1_{init}$	0.001	0.001	0.001	a.u.	Fixed initial effective concentration 1
$CONC2_{init}$	0.009	0.149	0.042	a.u.	Initial effective concentration 2
$CONC3_{init}$	0.032	0.655	0.166	a.u.	Initial effective concentration 3
$CONC4_{init}$	0.046	1.048	0.237	a.u.	Initial effective concentration 4
$CONC5_{init}$	0.07	2.455	0.374	a.u.	Initial effective concentration 5
$CONC6_{init}$	0.113	999.981	0.837	a.u.	Initial effective concentration 6
ER_{init}	0.026	0.467	0.126	a.u.	Initial ERα concentration
$GREB1_{init}$	0.834	0.915	0.913	a.u.	Initial GREB1 concentration
PR_{init}	0.781	0.789	0.794	a.u.	Initial PR concentration
$TFF1_{init}$	0.959	0.971	0.967	a.u.	Initial TFF1 concentration
$E2\_ER_{init}$	0	0	0	a.u.	Fixed initial E2-ERα complex concentration
$E2\_ER\_GREB1_{init}$	0	0	-	a.u.	Fixed initial E2-ERα/GREB1 concentration
$E2\_ER\_PR_{init}$	-	-	0	a.u.	Fixed initial E2-ERα/PR concentration
$E2\_ER\_PR\_GREB1_{init}$	-	-	0	a.u.	Fixed initial E2-ERα/PR/GREB1 concentration
d_{ER}	0.028	0.035	0.022	h^{-1}	ERα degradation rate
d_{PR}	0.022	0.010	0.066	h^{-1}	PR degradation rate
d_{GREB1}	0.155	0.287	0.373	h^{-1}	GREB1 degradation rate
d_{TFF1}	0.652	0.000	0.000	h^{-1}	TFF1 degradation rate
b_{E2_ER}	7.487	0.351	1.000	h^{-1}	Maximal binding rate of E2 with ERα
d_{E2_ER}	0.359	0.000	0.246	h^{-1}	Degradation rate of E2-ERα
$b_{E2\_ER\_GREB1}$	0.037	0.431	-	h^{-1}	Maximal binding rate of E2-ERα with GREB1
$d_{E2\_ER\_GREB1}$	0.000	0.018	-	h^{-1}	Degradation rate of E2-ERα/GREB1
$b_{E2\_ER\_PR}$	-	-	0.025	h^{-1}	Maximal binding rate of E2-ERα with PR
$d_{E2\_ER\_PR}$	-	-	0.814	h^{-1}	Degradation rate of E2-ERα/PR
$b_{E2\_ER\_PR\_GREB1}$	-	-	1.000	h^{-1}	Maximal binding rate of E2-ERα/PR with GREB1
$d_{E2\_ER\_PR\_GREB1}$	-	-	0.005	h^{-1}	Degradation rate of E2-ERα/PR/GREB1
$stim_{PR}$	63.502	0.363	67.697	a.u. · h^{-1}	Maximal stimulation rate of PR
$stim_{GREB1}$	673.314	8.434	1000.0	a.u. · h^{-1}**	Maximal stimulation rate of GREB1
$stim_{TFF1}$	952.197	2.874	10.470	h^{-1}***	Maximal stimulation rate of TFF1
s_{ER}	0.001	0.016	0.003	a.u. · h^{-1}	Calculated ERα basal synthesis rate (Eq. 18)
s_{PR}	0.017	0.008	0.052	a.u. · h^{-1}	Calculated PR basal synthesis rate (Eq. 19)
s_{GREB1}	0.129	0.263	0.340	a.u. · h^{-1}	Calculated GREB1 basal synthesis rate (Eq. 20)
s_{TFF1}	0.625	0.000	0.000	a.u. · h^{-1}	Calculated TFF1 basal synthesis rate (Eq. 21)
**Cell cycle model parameters**					
**Parameter**	**Value**			**Unit**	**Description**
	**Model I**	**Model II**	**Model III**		
a1	-	-	0.0018	a.u. · h^{-1}	Fixed CDK1 basal synthesis rate
a2	-	-	0.273	h^{-1}	Fixed APC synthesis rate
a3	-	-	0.273	h^{-1}	Fixed CDK1 autostimulatory synthesis rate
b1	-	-	0.273	h^{-1}	Fixed CDK1 degradation rate
b2	-	-	0.091	$a.u.^{-2} \cdot h^{-1}$	Fixed APC degradation rate
K1	-	-	0.5	a.u.	Fixed half-saturation constant 1
K2	-	-	0.5	a.u.	Fixed half-saturation constant 2
K3	-	-	0.5	a.u.	Fixed half-saturation constant 3
n1	-	-	8	-	Fixed Hill coefficient 1
n2	-	-	8	-	Fixed Hill coefficient 2
n3	-	-	8	-	Fixed Hill coefficient 3
$CDK1_{init}$	-	-	0.124	a.u.	Initial CDK1 concentration obtained by simulation
APC_{init}	-	-	0.526	a.u.	Initial APC concentration obtained by simulation
k_{GREB1}	-	-	0.0001	h^{-1}	Fixed GREB1-dependent CDK1 synthesis rate
r	-	-	2.2	-	Fixed multiplication factor

* Estimated values unless stated otherwise

** Unit h^{-1} in Model II

*** Unit a.u. · h^{-1} in Model I

Supplementary Table 2. Values and descriptions of the estimated degradation parameters in starvation medium without E2 exposure (see Equations 24-26).

**Parameter**	**Value**	**Unit**	**Description**
d_{PR}	0.0095	h^{-1}	PR degradation rate
d_{GREB1}	0.0191	h^{-1}	GREB1 degradation rate
d_{TFF1}	0.0216	h^{-1}	TFF1 degradation rate
